# Administration of enteral nutrition in the prone position, gastric residual volume and other clinical outcomes in critically ill patients: a systematic review

**DOI:** 10.5935/0103-507X.20200019

**Published:** 2020

**Authors:** Letiane de Souza Machado, Paula Rizzi, Flávia Moraes Silva

**Affiliations:** 1 Multidisciplinary Residency Program in Health: Emphasis on Intensive Care, Universidade Federal de Ciências de Saúde de Porto Alegre - Porto Alegre (RS), Brazil.; 2 Department of Nutrition, Universidade Federal de Ciências de Saúde de Porto Alegre - Porto Alegre (RS), Brazil.; 3 Postgraduate Program in Nutrition Sciences, Universidade Federal de Ciências de Saúde de Porto Alegre - Porto Alegre (RS), Brazil.

**Keywords:** Nutrition therapy, Enteral nutrition, Prone position, Critical illness, Gastrointestinal contents, Pneumonia, Terapia nutricional, Nutrição enteral, Posição prona, Paciente crítico, Conteúdo gastrointestinal, Pneumonia

## Abstract

This systematic review of longitudinal studies aimed to evaluate the effect of enteral feeding of critically ill adult and pediatric patients in the prone position on gastric residual volume and other clinical outcomes. A literature search was conducted in the databases PubMed, Scopus and Embase using terms related to population and intervention. Two independent reviewers analyzed the titles and abstracts, and data collection was performed using a standardized form. Discrepancies were resolved by a third reviewer. The methodological quality of the studies was evaluated considering the potential for systematic errors, and the data were qualitatively analyzed. Four studies with adult patients and one with preterm patients were included. The gastric residual volume was evaluated as the main outcome: three studies did not show differences in the gastric residual volume between the prone and supine positions (p > 0.05), while one study showed a higher gastric residual volume during enteral feeding in the prone position (27.6mL *versus* 10.6mL; p < 0.05), and another group observed a greater gastric residual volume in the supine position (reduction of the gastric residual volume by 23.3% in the supine position *versus* 43.9% in the prone position; p < 0.01). Two studies evaluated the frequency of vomiting; one study found that it was higher in the prone position (30 *versus* 26 episodes; p < 0.001), while the other study found no significant difference (p > 0.05). The incidence of aspiration pneumonia and death were evaluated in one study, with no difference between groups (p > 0.05). The literature on the administration of enteral feeding in the prone position in critically ill patients is sparse and of limited quality, and the results regarding gastric residual volume are contradictory. Observational studies with appropriate sample sizes should be conducted to support conclusions on the subject.

## Abstract (pt)

Esta revisão sistemática de estudos longitudinais objetivou avaliar o efeito da administração da dieta enteral em pacientes críticos adultos e pediátricos em posição prona no volume residual gástrico e em outros desfechos clínicos. A busca da literatura foi conduzida nas bases de dados PubMed®, Scopus e Embase, a partir de termos relacionados à população e à intervenção. Dois revisores independentes analisaram os títulos e resumos, e a coleta dos dados foi realizada a partir de uma ficha padronizada. Discrepâncias foram resolvidas por um terceiro revisor. A qualidade metodológica dos estudos foi avaliada considerando o potencial para erros sistemáticos e os dados analisados qualitativamente. Quatro estudos com pacientes adultos e um com pacientes pré-termos foram incluídos. O volume residual gástrico foi avaliado como principal desfecho: três não diferiram no volume residual gástrico entre as posições prona e supina (p > 0,05), enquanto um estudo demonstrou maior volume residual gástrico durante a administração da dieta em posição prona (27,6mL versus 10,6mL; p < 0,05), e outro apresentou maior volume residual gástrico na posição supina (redução do volume residual gástrico de 23,3% na posição supina versus 43,9% na posição prona; p < 0,01). Dois estudos avaliaram a frequência de vômitos, sendo maior na posição prona em um estudo (30 versus 26 episódios; p < 0,001) e sem diferença significativa em outro (p > 0,05). Incidência de pneumonia aspirativa e de óbito foram avaliadas por um estudo, não sendo observada diferença entre os grupos (p > 0,05). A literatura acerca da administração de dieta enteral em pacientes críticos em posição prona é escassa e de qualidade limitada, e os resultados sobre volume residual gástrico são contraditórios. Estudos observacionais com tamanho amostral apropriado deveriam ser conduzidos para fundamentar conclusões sobre o tema.

## INTRODUCTION

Acute respiratory distress syndrome (ARDS) is a type of inflammatory lung injury caused by increased pulmonary vascular permeability, the clinical effects of which are hypoxemia and noncardiogenic pulmonary edema.^(1)^ According to a cohort study conducted in the intensive care units (ICUs) of 21 hospitals in the United States, the incidence rate of ARDS was 58 cases/100,000, with an estimated mortality rate of 25 - 40%.^(2)^

The prone position (PP) is defined as the turning of the patient from the supine position (SP) to ventral decubitus, which allows better expansion of the dorsal lung regions with consequent improvement in oxygenation.^(3)^ A review of 31 studies concluded that the prone positioning of patients with ARDS can lead to an oxygenation improvement of approximately 70 - 80%, which partially persists after the patient is switched back to the SP; additionally, PP does not affect the respiratory mechanics and rarely causes complications.^(4)^ A meta-analysis of eight randomized clinical trials showed a 26% reduction in the incidence of death in the subgroup of studies that maintained PP for at least 12 hours in patients with moderate or severe ARDS.^(5)^ Bloomfield et al. also showed that longer times in PP had benefits for more hypoxemic patients in a meta-analysis of nine primary studies.^(6)^ In fact, the effect of PP on mortality seems to be particularly evident in patients with a partial pressure of oxygen/fraction of inspired oxygen (PaO_2_:FIO_2_) ratio < 150.^(7,8)^

Although its validity is widely described in the literature, the use of PP is not very common in the ICU: data from the literature indicate that the use of this protocol ranges from 2.8% to 16.3% for patients with severe ARDS.^(9^^.^^10)^ In addition to the infrequent use of PP, several studies in the literature that evaluated the effect of this positioning on the clinical outcomes of patients with ARDS do not provide any description of nutritional therapy in the prone positioning protocols.^(10-12)^ A study of 51 critically ill patients reported discontinuation of enteral nutritional therapy (ENT) in 25% of the sample during the pronation period.^(13)^ In addition, a retrospective study of critically ill patients with ARDS that applied the prone positioning protocol found that the administration of enteral feeding in the PP was insufficient in 82.9% of the patients,^(14)^ which contributed to a negative energy and protein balance. Negative energy and protein balance in critically ill patients is associated with an increase in the number of complications, particularly infections, duration of mechanical ventilation, length of hospital stay^(15)^ and mortality.^(16)^

Considering the relevance of the topic, the aim of the present study was to systematically review the scientific literature on the effect of administering enteral nutrition (EN) in the PP on the gastric residual volume (GRV) and clinical outcomes of critically ill adult and pediatric patients.

## METHODS

### Design

A systematic review of longitudinal studies conducted according to the Cochrane Collaboration recommendations^(17)^ and presented according to the Preferred Reporting Items for Systematic Reviews and Meta-Analyses (PRISMA) recommendations.^(18)^

### Research question

The research question of the present systematic review was elaborated according to the PICO strategy: Does the administration of EN in critically ill adult and pediatric patients (P = population) in the PP (I = intervention) increase the GRV and the risk of worse clinical outcomes (O = outcome) compared to the administration of enteral feeding in SP (C = control)?

### Inclusion and exclusion criteria

Longitudinal studies comparing the effect of enteral feeding on critically ill adult and pediatric patients in the PP and SP on the GRV and/or on the incidence of aspiration pneumonia and other clinical outcomes were selected.

Studies that were performed on patients who were not admitted to the ICU and on those who were not on ventilatory support were excluded from the present systematic review. Additionally, descriptive studies, reviews and unpublished studies were not included.

### Search strategies

The literature search was performed in three databases (PubMed, Scopus and Embase) in April 2018 with indexing terms related to EN and PP. The main MesH employed in the search were the following: “Nutrition Therapy”, “Nutritional Support”, “Enteral Nutrition” and “Prone Position”. No restrictions were applied regarding the language or date of publication. The search strategy used in PubMed® is shown in table 1. The search was updated in October 2018.

**Table 1:** Search strategy employed in the PubMed^®^ database

Nutrition Therapy[Title/Abstract]) OR Nutrition Therapy, Medical[Title/Abstract]) OR Therapy, Medical Nutrition[Title/Abstract]) OR Support, Nutritional[Title/ Abstract]) OR Nutritional Support[Title/Abstract]) OR Artificial Feeding[Title/ Abstract]) OR Feeding, Artificial[Title/Abstract])) OR ((((((((((((((Enteral Feeding[Title/Abstract]) OR Feeding, Enteral[Title/Abstract]) OR Force Feeding[Title/Abstract]) OR Feeding, Force[Title/Abstract]) OR Feedings, Force[Title/Abstract]) OR Force Feedings[Title/Abstract]) OR Tube Feeding[Title/ Abstract]) OR Feeding, Tube[Title/Abstract]) OR Gastric Feeding Tubes[Title/ Abstract]) OR Feeding Tube, Gastric[Title/Abstract]) OR Feeding Tubes, Gastric[Title/Abstract]) OR Gastric Feeding Tube[Title/Abstract]) OR Tube, Gastric Feeding[Title/Abstract]) OR Tubes, Gastric Feeding[Title/Abstract]))) AND (((((Prone position[Title/Abstract]) OR Position, Prone[Title/Abstract]) OR Positions, Prone[Title/Abstract]) OR Prone Positions[Title/Abstract]) OR Pronation[Title/Abstract])

### Selection of studies

The studies identified in the databases were organized in a library using the reference management software EndNote®, and duplicates were excluded. The eligibility of the studies was evaluated based on the following criteria: patients receiving EN, mechanical ventilation and the prone positioning protocol.

The selection of eligible studies was performed in two phases. In the first phase, two independent reviewers read the titles and abstracts of all selected articles, and in the second phase, the full texts were read. In both phases, disagreements were resolved by a third reviewer.

### Data collection

After the selection of studies that were eligible for the present systematic review, data collection was independently performed by two reviewers using a standardized data collection form, which included information about the publication (year, journal, author and country), study sample (age, sex and severity), study (design and follow-up time), interventions of interest (characteristics of the enteral feeding and the prone positioning protocol adopted) and analyzed outcomes.

The outcomes of interest were not established *a priori* except for GRV and the incidence of aspiration pneumonia. Consequently, all other outcomes analyzed by the authors of the primary studies, when present, were collected. These outcomes included other indicators of intolerance of EN (such as vomiting and the cessation of enteral feeding) and mortality. Disagreements in the data collection process were resolved through the reading of the full text by the third reviewer.

### Assessment of the risk of bias

Considering that the eligible studies had different designs (including before-and-after studies and observational comparative studies), their methodological quality could not be evaluated using the tools recommended by the Cochrane Collaboration.^(17)^ However, some criteria were evaluated to weigh the risk of systematic errors and the precision of the results, as detailed below:

1. Precision: analyzed by the width of the 95% confidence interval (95% CI), or, in the absence of this, the sample size of the studies.

2. Risk of systematic error: analyzed based on the assessment of risk of measurement bias and confounding bias, considering the methodology described by the authors. We also considered whether the authors performed multivariate analysis with adjustment for potential confounders.

### Synthesis and analysis of data

Considering the heterogeneity of the studies as a result of differences in designs, participants characteristics (age and severity), the prone positioning protocols used, the type of EN administered and the diversity of outcomes analyzed, it was not possible to conduct a meta-analysis to obtain a weighted estimate of the effect of EN administration in the PP compared to SP and/or other positions on the outcomes of interest. Therefore, the data were synthesized based on a qualitative evaluation.

## RESULTS

### General characteristics of the studies

**Figure 1:**
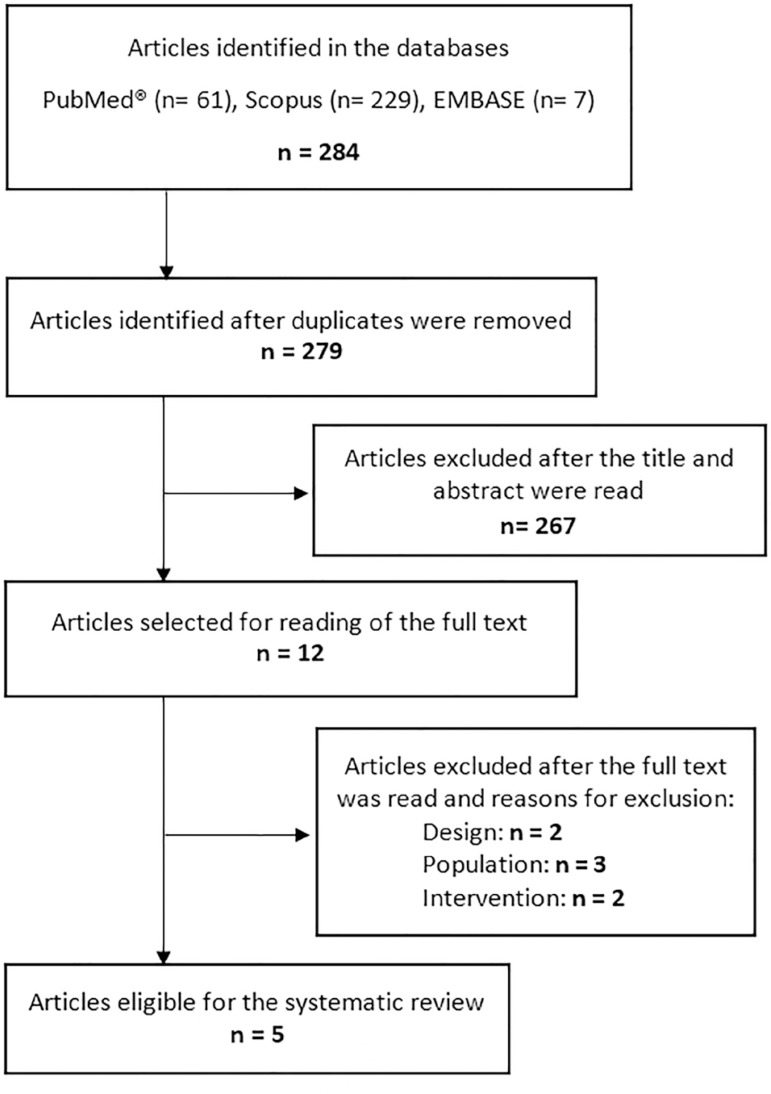
Flowchart of study selection.

The flowchart of the selection of studies for the present systematic review is shown in figure 1. Five studies were included in the present systematic review;^(9,19-22)^ four were conducted with adult patients^(9,19,21,22)^, and one was conducted with preterm pediatric patients.^(20)^ One study was conducted in France,^(19)^ one in Spain,^(9)^ one in Taiwan,^(20)^ one in Italy^(22)^ and another in the Netherlands.^(21)^

All studies had an observational design; four were prospective.^(9,19-22)^ One study had a retrospective design.^(22)^ Only one study included sample size calculation.^(20)^ The follow-up time was described in four studies and ranged between 8.5 hours^(20)^ and 24.7 days; ^(9)^ it was not described in the other study.^(22)^

The number of participants in the primary studies ranged from 19^(21)^ to 71.^(19)^ In most of the studies,^(9,19,21,22)^ all patients were on mechanical ventilation (MV); the exception was the study performed on preterm patients, in which the participants were on MV or noninvasive ventilation.^(20)^

Among the studies conducted with adult patients,^(9,19,21,22)^ the mean age was 55.4 years, ranging from 47.6 years^(9)^ to 65.1 years.^(21)^ The mean age of the patients in the study conducted with pediatric patients was 29.7 weeks.^(20)^ The proportion of male participants among the studies was 63.02%, with a minimum of 58%^(9)^ and a maximum of 73.7%,^(21)^ This information was not provided in one study.^(22)^

The other general characteristics of the studies on the effect of EN provided in the PP for critically ill patients are described in table 2.

**Table 2:** General characteristics of studies on the administration of enteral nutrition in the prone position for critically ill patients

Author, location	Design, follow-up	Sample	Interventions under study	Outcomes of interest
Saez de la Fuente et al.^(9)^	Prospective observational study Duration: 24.7 ± 12.3 days under ENT	n = 34 Adult patients on MV with prescription of ENT in prone position Age: 47.6 ± 18.4 years Men: 58% APACHE II: 33.3% of the sample between 10 and 14 points	Control: supine position, with interruption of enteral nutrition if GRV > 500 mL in 6 hours. Enteral feeding via infusion pump for 24 hours. Initial volume of 25% of the nutritional target achieved in 96 hours Intervention: prone position, if severe hypoxemia (PaO_2_/FiO_2_ < 150) in the presence of hemodynamic stability for 48 consecutive hours; neck/head were alternated to the right and left every 2 hours. Enteral feeding via infusion pump for 24 hours. Initial volume of 25% of the nutritional target was reached in 96 hours. Angle of elevation of the bed of 10º in reverse Trendelenburg	GRV (measured every 6 hours) Vomiting Regurgitation
Reignier et al.^(19)^ França	Prospective observational study Duration: 5 days	n = 71 (37 control/34 intervention) Adult clinical patients on invasive and MV and sedated with the prediction of ENT for at least 5 days Age: 58 ± 16.5 years Men: 71.8% SAPS II: 52 ± 22 points	Control: semirecumbent supine position. Enteral feeding via infusion pump for 18 hours. Initial volume of 30 mL/hour; progression to nutritional target in 96 hours Intervention: prone position in cases of severe hypoxemia (PaO_2_/FiO_2_ < 150; FiO_2_ = 0.6; PEEP = 10 cmH2O). Enteral feeding via infusion pump for 18 hours (6-hour rest in the supine or prone position, with position determined randomly). Initial volume of 30 mL/hour and progression to nutritional target in 96 hours. Elevated head of the bed	GRV (measured every 6 hours) Vomiting MV-associated pneumonia Mortality
Chen et al.^(20)^ Taiwan	Crossover randomized series Duration: 8.5 hours	n = 35 Convenience sample of preterm infants with Apgar score > 7 who were receiving ENT and were clinically stable Age: 29.75 ± 3.01 weeks Boys: 48.6%	Participants were allocated to two groups: in one group, the sequence of positions was supine-prone, and in the other group, the sequence was prone-supine. Subsequently, the order was inverted. In both groups, BM was administered via OGT by infusion pump. The initial volume was 20 mL/kg/day, with volume increases every 3 hours to 160 mL/kg/day of nutritional target. Two stages of BM administration: 50 mL/kg/day and 100 mL/kg/day	GRV (measured 30, 60, 90, 120 and 150 minutes after BM infusion)
Van der Voort et al.^(21)^ Holanda	Prospective observational study Duration: 12 hours	n = 19 Adult patients on MV in the prone position with onset of ENT during the first 24 hours of ICU stay Age: 65.1 (41 - 82) years Men: 73.7% Apache II: 25.5 ± 8.9 points	Control: supine position for 6 hours, with head of the bed elevated at 30°. Enteral feeding 80 mL/hour as nutritional target Intervention: prone position if hypoxemia (PaO_2_/FiO_2_ < 100) or pneumonia with excessive production of bronchial secretion - maintained for 6 hours, after patient is placed in supine position. Head of the bed elevated at 30°. Enteral feeding 80 mL/hour as the nutritional target*	GRV (measured every 6 hours)
Lucchini et al.^(22)^ Itália	Retrospective observational study Information about follow-up period not provided	n = 25 patients with ARDS on MV and continuous ENT Age: 51.13 ± 15.93 years Men: not stated RASS: median of -5 in both groups (p = 0.165)	Control: supine position with head of the bed elevated to at least 15º Intervention: prone position with head of the bed elevated to at least 15º (pronation criteria were not described) Enteral nutrition interrupted if GRV > 300 mL, administration of metoclopramide and return to previous volume in both groups	GRV (measured every 3 hours)

ENT - Enteral nutritional therapy; MV - mechanical ventilation; APACHE II - Acute Physiology and Chronic Health Evaluation II; GRV - gastric residual volume; PaO_2_ - arterial pressure of oxygen; FiO_2_ - fraction of inspired oxygen; SAPS II - Simplified Acute Physiology Score II; PEEP - positive end-expiratory pressure; BM - breast milk; OGT - orogastric tube; ICU - intensive care unit; ARDS - acute respiratory distress syndrome; RASS - Richmond Agitation and Sedation Scale.

* Prokinetics were not administered during the study.

### The prone positioning protocols and nutritional therapy monitoring protocols used

The prone positioning protocols differed among the studies and were not clearly described in one of the studies,^(20)^ as shown in table 2. The prone positioning criteria were described in three studies,^(9,19,21)^ among which two adopted the same criteria for the definition of severe hypoxemia. ^(9,19)^ In two studies, the authors describe elevating the head of the bed in the PP alone,^(9,19)^ whereas in two studies, the same angle of the head of the bed was used during both PP and SP,^(21,22)^ and in one study, this information was not described.^(20)^

The majority of the studies conducted with adult patients assessed GRV every 6 hours;^(9,19,21)^ the exception was one study in which the GRV was checked every 3 hours.^(22)^ The study conducted with preterm patients measured GRV every 30 minutes for 2 hours and 30 minutes after the administration of breast milk.^(20)^ GRV was adopted as a criterion for stopping ENT and/or introducing prokinetics, but the cutoff points adopted differed among the studies included in the present systematic review. The largest and smallest GRVs adopted for stopping EN were 500mL^(9)^ and 150mL,^(21)^ respectively. One study adopted a GRV > 300mL,^(22)^ and in another study, a GRV > 250mL^(19)^ was used as a cutoff for interrupting EN. In the study conducted with critically ill pediatric patients, the GRV adopted as a criterion for continuing administering EN was less than 50% of the pregavage GRV.^(20)^

Only one study did not consider the administration of prokinetics or facilitators of gastric emptying when ENT intolerance was evidenced (i.e., in cases of nausea, vomiting or elevated GRVs).^(21)^

### Effect of the administration of enteral nutrition in the prone position for critically ill patients on gastric residual volume and other clinical outcomes

The results of the studies on the effect of enteral feeding in the PP for critically ill patients are described in table 3. All the studies included in the present systematic review evaluated GRV as the main outcome: in three studies,^(9,21,22)^ the mean GRV did not differ significantly during the administration of EN in PP compared to the SP. In one study, the mean GRV at 5 days of monitoring was significantly higher in the PP than in the SP [10 (0 - 58.6) *versus* 27.6 (3.8 - 119.4) mL].^(19)^ In contrast, in another study conducted with newborns, the reduction in GRV was significantly higher in the PP compared to the SP (43.95% *versus* 23.26%, for a 50 mL/kg/day infusion volume, and 48.07% *versus* 28.46% for a 100mL/kg/day infusion volume).^(20)^

**Table 3:** Effect of enteral nutrition in critically ill patients administered in prone position compared to supine position on clinical outcomes

Author	Intolerance of ENT	Aspiration pneumonia	Other outcomes	Conclusion	Strengths and weaknesses
Saez de la Fuente et al.^(9)^	GRV/day (p = 0.054): Control: 126.6 ± 132.1 mL Intervention: 189.2 ± 203.2 mL Frequency of elevated GRV/day (p = 0.39): Control: 0.06 ± 0.01 Intervention: 0.09 ± 0.17 Frequency of vomiting/day (p = 0.53): Control: 0.016 ± 0.03 Intervention: 0.03 ± 0.09 Regurgitation/day (p =0.051): Control: 0 Intervention: 0.04 ± 0.13	Not evaluated	Not evaluated	ENT in critically ill patients with severe hypoxemia in prone position is viable and safe and is not associated with an increase in gastrointestinal complications	Weaknesses: Duration of ENT during supine position significantly longer than the prone position Sample size not calculated Enteral nutrition volume administered differed during prone and supine position. Strengths: Crossover design
Reignier et al.^(19)^	GRV (mean of 5 days): Control: 10.6 (0 - 58.6)mL Intervention: 27.6 (3.8 - 119.4)mL Significant difference at days 1, 2 and 4 (p <0.01) Interruption of EN (p < 0.01): Control: 49% Intervention: 82% Vomiting (p < 0.001): Control: 26 episodes Intervention: 30 episodes RR = 2.5 (1.5 - 4.0)	Associated with VM (NS): Control: 24% Intervention: 35%	Infused volume (mean of 5 days): Control: 1095 (876 - 1336) mL Intervention: 754 (552 - 929) mL Significant difference in the five days (p <0.05). Mortality (NS): Control: 24% Intervention: 35%	In severely hypoxemic patients on invasive MV, the administration of EN in the prone position is associated with a higher frequency of vomiting	Weaknesses: Allocation of the subjects to the groups according to the need for pronation Regular measurement of gastric residual volume and vomiting may underestimate changes in gastric emptying and esophageal reflux No sample size calculation was presented. Strengths: Similar groups regarding age, gender and SAPS (minimizes confounding bias)
Chen et al.^(20)^	Redução % do VRG: 1º estágio - 50mL/kg/dia (p < 0,01): Controle: 23,26% Intervenção: 43,95% 2º estágio - 100mL/kg/dia (p < 0,01): Controle: 28,46% Intervenção: 48,07%	Not evaluated	Not evaluated	Preterm infants have lower GRV in the prone position compared to the supine position, when 50 mL/kg/day and 100 mL/kg/day are offered	Weaknesses: Short-term GRV assessment Breast milk may limit the generalization of results Fixed volumes of breast milk administered Strengths: Sample size calculated Random allocation Allocation Blinding
Van der Voort et al.^(21)^	VRG em 3 horas (p = 0,69): Controle: 59,5 (0 - 180) mL Intervenção: 59,7 (0 - 200) mL VRG em 6 horas (p = 0,85): Controle: 110 (0 - 325) mL Intervenção: 95 (10 - 340) mL	Not evaluated	Not evaluated	GRV did not differ significantly after 3 and 6 hours of enteral nutrition in the prone or supine position	Weaknesses: Sample size not calculated Order of interventions was not randomized Does not inform method of administration of the ENT and initial volume Short duration of interventions Strengths: Constant EN volume Crossover design
Lucchini et al.^(22)^	VRG (p = 0,73): Controle: 20,62 ± 18,92mL I: 23,62 ± 50,02mL VRG > 300 ml (p = 0,65): Controle: 2 (0,4%) I: 2 (0,8%)	Not evaluated	Not evaluated	The administration of ENT in the prone position did not promote a significant increase in GRV compared to the supine position	Weaknesses: Sample size Criteria for pronation not described Strengths: Infused volume did not differ between groups

ENT - Enteral nutritional therapy; GRV - gastric residual volume; EN - enteral nutrition; RR - relative risk; MV - mechanical ventilation; NS - not significant; SAPS - Simplified Acute Physiology Score.

The frequency of vomiting was analyzed in two studies.^(9,19)^ In one study, the frequency of vomiting was significantly higher in PP compared to SP, and PP was associated with a 2.5-fold increase (95% CI 1.5 - 4.0) in the odds of patients exhibiting vomiting.^(19)^ In another study, the daily number of episodes and the number of regurgitations did not differ between PP and SP.^(9)^

Only one study evaluated the incidence of aspiration pneumonia and death. No significant difference was observed in the results of PP and SP, although ventilator-associated pneumonia and the occurrence of death were observed in 35% of the patients in PP and 24% of the patients in SP.^(19)^

### Methodological quality of the studies: risk of bias and precision of the results

Table 4 shows the risk of systematic errors and the precision of the findings of the studies included in the present systematic review as well as the justification for the classification provided. Most of the studies presented results with low precision^(9,19,21,22)^ due to the reduced sample size and the wide confidence interval of the results. The risk of measurement bias^(9,19-21)^ was low in most studies, while the risk of confounding bias was uncertain in most of them.^(19,21,22)^ None of the studies presented the results of multivariate analysis with adjustment for potential confounders.

**Table 4:** Methodological quality of studies on enteral nutrition administration in the prone position for critically ill patients: precision and risk of bias

Author	Precision	Measurement bias	Confounding bias/multivariate analysis
Saez de la Fuente et al.^(9)^	High risk Wide confidence interval for the result Sample size not calculated	Low risk Adequate measurement of the factors under study and outcomes	High risk Duration of supine position longer than that of the prone position and the volume of enteral feeding administered differed between groups Multivariate analysis not performed
Reignier et al.^(19)^	High risk Wide confidence interval for the result Sample size not calculated	Low risk Adequate measurement of the factors under study and outcomes	Uncertain risk Similar characteristics at baseline Multivariate analysis not performed
Chen et al.^(20)^	Low risk Sample size calculated in a pilot study	Low risk Adequate measurement of the factors under study and outcomes	Low risk Random allocation of the order of interventions, crossover design Multivariate analysis not performed
Van der Voort et al.^(21)^	High risk Wide confidence interval for the result Sample size not calculated	Low risk Adequate measurement of the factors under study and outcomes	Uncertain risk Criteria for sample selection not clearly described Despite nonrandom allocation of the order of interventions, crossover design Multivariate analysis not performed
Lucchini et al.^(22)^	High risk Wide confidence interval for the result Sample size not calculated	Uncertain risk Criteria for pronation were not described	Uncertain risk Unclear whether it was a crossover design or whether the allocation was random. Multivariate analysis not performed

The weaknesses and strengths of each study are reported in table 3. Common weaknesses of most studies were a reduced sample size^(9,20-22)^ and a short outcome assessment period.^(20,21)^

## DISCUSSION

The aim of the present systematic review was to evaluate the effect of EN in the PP in critically ill patients on gastrointestinal tolerance and clinical outcomes; five eligible observational studies were identified. The results regarding the gastrointestinal tolerance of patients during pronation, which was evaluated mainly by measuring GRV, were contradictory. Furthermore, the incidence of aspiration pneumonia and death was evaluated in only one study, and there was no association with the positioning of patients during the administration of EN.

Patients with ARDS have a proinflammatory condition and marked protein catabolism, which can lead to an increase in daily energy expenditure of up to 20%.^(23)^ Adequate nutritional intake should be offered early to avoid the reduction of respiratory muscle strength, which can occur a few days after the onset of underfeeding.^(24)^ Nevertheless, the risk of aspiration due to gastrointestinal intolerance in these patients may be a concern related to the administration of EN during pronation. In fact, two studies included in the present systematic review concluded that in PP, early EN is poorly tolerated, as evidenced by a higher frequency of episodes of vomiting, greater discontinuation of EN, a lower rate of EN infusion ^(19)^ and a lower mean number of days receiving ENT.^(9)^ These findings corroborate the data reported in other studies: in a study involving 51 patients in pronation, discontinuation of EN was observed in 25% of the sample,^(13)^ and the administration of enteral feeding during PP was insufficient in 82.9% of the sample in a retrospective study conducted with critically ill clinical patients with ARDS.^(14)^

Only one study in the present systematic review was conducted with the pediatric population: in contrast with the findings for adults, premature infants had a lower GRV during PP compared to SP, especially in the first 30 minutes after EN administration. However, it should be noted that in that study, the patients received fixed EN volumes, and only breast milk was administered.^(20)^ Such particularities preclude the generalization of these results as many premature infants in the pediatric ICU do not receive breast milk, and the volumes vary according to the weight of the patients.

All of the studies included in the present systematic review used GRV to evaluate the tolerance of EN administered in the PP in critically ill patients. However, the applicability of GRV as a predictor of the incidence of aspiration pneumonia is not supported by the current scientific evidence, despite its routine use in clinical practice. In a multicenter randomized clinical trial involving 449 critically ill patients allocated to a group in which the GRV was monitored and routine measures were adopted to manage elevated GRV or to a group in which the GRV was not monitored, there was no significant difference between groups in the incidence of ICU-acquired infections, the duration of mechanical ventilation, the length of stay or the incidence of death.^(25)^ The American Society of Parenteral and Enteral Nutrition (ASPEN)^(26)^ and the European Society of Parenteral and Enteral Nutrition (ESPEN)^(27)^ recommend that continuous monitoring of GRV not be part of the routine care of critically ill patients and that, in units that still monitor this parameter, EN be delayed if the GRV is greater than 500mL/6 hours when other strategies have been adopted without positive results (e.g., positioning of the head of the bed, EN infusion rate, caloric density of the formula, medical prescription of prokinetics).^(26,27)^ The suggested cutoff point of 500 mL is justified by the results of a clinical trial that randomized critically ill patients on MV to a protocol of corrective strategies if GRV > 200 mL/6 hours or if GRV > 500mL/6 hours; this study did not show a significant difference in the incidence of aspiration pneumonia between the groups.^(28)^

The methodological quality of the studies included in the present systematic review cannot be evaluated using the scales recommended by the guidelines due to their designs. Therefore, the internal validity of the studies was evaluated based on the risk of systematic errors and the precision of the findings; additionally, a critical analysis of the articles highlighted the strengths and weaknesses of each study. In general, the studies presented questionable methodological quality, which compromises the validity of the findings. Ideally, longitudinal studies with adequate sample sized and long-term follow-up should be conducted so that the effects of EN administration in the PP on the clinical outcomes of critically ill patients can be better elucidated. In fact, a systematic review of the literature previously published by Linn et al. also showed that the evidence on the safety and tolerability of EN in pronated patients is quite limited. ^(29)^ The authors included four studies; two of these were eligible for the present review, while the other two did not intend to compare the outcomes associated with EN in PP *versus* SP and, therefore, did not meet our eligibility criteria.

Despite the limited scientific evidence, when a team chooses to administer EN in PP, a protocol to minimize the risk of intolerance should be developed and should include some minimum aspects, including elevating the head of the bed, using an enteral formula with a higher calorie density to reduce the volume infused per hour, providing continuous EN administration by infusion pump, using prophylactic prokinetics, and alternating the neck/head to the right and left every 2 hours. Furthermore, the progression of EN to the nutritional target should be slow, starting at approximately 25% and reaching the total volume to be infused in 96 hours.^(9,21,22)^ It is noteworthy that the combination of a greater number of care measures listed above may offer greater protection against EN intolerance. According to the checklist for safe pronation recently proposed by Oliveira et al., nutritional care during the protocol should include the following: (1) positioning of an enteral feeding tube in a postpyloric position with radiological confirmation; (2) head of the bed elevated to a 25-30° angle (reverse Trendelenburg); (3) prescription of a fixed prokinetic agent (erythromycin 250mg intravenously every 6 hours); (4) early diet after the first hour, with 30 ml/hour administered until the sixth hour, 40mL/hour administered from the sixth to the 12^th^ hour and 50mL/hour administered until 1 hour before the return to the SP.^(8)^ According to ESPEN, ENT must be instituted early, even in patients in the PP.^(27)^

## CONCLUSION

The available literature on the effect of enteral nutrition administered in the prone position for critically ill patients on gastrointestinal tolerance and clinical outcomes is scarce and has limited methodological quality; therefore, it is not possible to establish any conclusions about the safety and/or benefits/adverse effects of this procedure. Long-term prospective studies with longer follow-up times and larger sample sizes are necessary to better analyze these aspects.

## References

[1] Ranieri VM, Rubenfeld GD, Thompson BT, Ferguson ND, Caldwell E, Fan E, ARDS Definition Task Force (2012). Acute respiratory distress syndrome: the Berlin definition. JAMA.

[2] Rubenfeld GD, Caldwell E, Peabody E, Weaver J, Martin DP, Neff M (2005). Incidence and outcomes of acute lung injury. N Engl J Med.

[3] Gattinoni L, Tognoni G, Pesenti A, Taccone P, Mascheroni D, Labarta V, Malacrida R, Di Giulio P, Fumagalli R, Pelosi P, Brazzi L, Latini R, Prone-Supine Study Group (2001). Effect of prone positioning on the survival of patients with acute respiratory failure. N Engl J Med.

[4] Pelosi P, Brazzi L, Gattinoni L (2002). Prone position in acute respiratory distress syndrome. Eur Respir J.

[5] Munshi L, Del Sorbo L, Adhikari NK, Hodgson CL, Wunsch H, Meade MO (2017). Prone position for acute respiratory distress syndrome: A systematic review and meta-analysis. Ann Am Thorac Soc.

[6] Bloomfield R, Noble DW, Sudlow A (2015). Prone position for acute respiratory failure in adults. Cochrane Database Syst Rev.

[7] Associação de Medicina Intensiva Brasileira, Sociedade Brasileira de Pneumologia e Tisiologia (2013). Diretrizes Brasileiras de Ventilação Mecânica.

[8] Oliveira VM, Piekala DM, Deponti GN, Batista DC, Minossi SD, Chisté M (2017). Checklist da prona segura: construção e implementação de uma ferramenta para realização da manobra de prona. Rev Bras Ter Intensiva.

[9] Saez de la Fuente I, Saez de la Fuente J, Quintana Estelles MD, Garcia Gigorro R, Terceros Almanza LJ, Sanchez Izquierdo JA (2016). Enteral nutrition in patients receiving mechanical ventilation in a prone position. JPEN J Parenter Enteral Nutr.

[10] Bellani G, Laffey JG, Pham T, Fan E, Brochard L, Esteban A, Gattinoni L, van Haren F, Larsson A, Mcauley DF, Ranieri M, Rubenfeld G, Thompson BT, Wrigge H, Slutsky AS, Pesenti A, LUNG SAFE InvestigatorsESICM Trials Group (2016). Epidemiology, patterns of care, and mortality for patients with acute respiratory distress syndrome in intensive care units in 50 countries. JAMA.

[11] Fernandez R, Trenchs X, Klamburg J, Castedo J, Serrano JM, Besso G (2008). Prone positioning in acute respiratory distress syndrome: a multicenter randomized clinical trial. Intensive Care Med.

[12] Guérin C, Reignier J, Richard JC, Beuret P, Gacouin A, Boulain T, Mercier E, Badet M, Mercat A, Baudin O, Clavel M, Chatellier D, Jaber S, Rosselli S, Mancebo J, Sirodot M, Hilbert G, Bengler C, Richecoeur J, Gainnier M, Bayle F, Bourdin G, Leray V, Girard R, Baboi L, Ayzac L, PROSEVA Study Group (2013). Prone positioning in severe acute respiratory distress syndrome. N Engl J Med.

[13] L'Her E, Renault A, Oger E, Robaux MA, Boles JM (2002). A prospective survey of early 12-h prone positioning effects in patients with the acute respiratory distress syndrome. Intensive Care Med.

[14] Jové Ponseti E, Villarrasa Millán A, Ortiz Chinchilla D (2017). Análisis de las complicaciones del decúbito prono en el síndrome de distrés respiratorio agudo: estándar de calidad, incidencia y factores relacionados. Enferm Intensiva.

[15] Villet S, Chiolero RL, Bollmann MD, Revelly JP, Cayeux RN MC, Delarue J (2005). Negative impact of hypocaloric feeding and energy balance on clinical outcome in ICU patients. Clin Nutr.

[16] Allingstrup MJ, Esmailzadeh N, Wilkens Knudsen A, Espersen K, Hartvig Jensen T, Wiis J Provision of protein and energy in relation to measured.

[17] Higgins JP, Green S Cochrane Handbook for Systematic Reviews of Interventions 5.1.0.

[18] Moher D Altman DG, PRISMA Group (2009). Preferred reporting items for systematic reviews and meta-analyses: the PRISMA statement. Ann Intern Med.

[19] Reignier J, Thenoz-Jost N, Fiancette M, Legendre E, Lebert C, Bontemps F (2004). Early enteral nutrition in mechanically ventilated patients in the prone position. Crit Care Med.

[20] Chen SS, Tzeng YL, Gau BS, Kuo PC, Chen JY (2013). Effects of prone and supine positioning on gastric residuals in preterm infants: a time series with crossover study. Int J Nurs Stud.

[21] van der Voort PH, Zandstra DF (2001). Enteral feeding in the critically ill: comparison between the supine and prone positions: a prospective crossover study in mechanically ventilated patients. Crit Care.

[22] Lucchini A, Bonetti I, Borrelli G, Calabrese N, Volpe S, Gariboldi R (2017). Enteral nutrition during prone positioning in mechanically ventilated patients. Assist Inferm Ric.

[23] Cerra FB, Benitez MR, Blackburn GL, Irwin RS, Jeejeebhoy K, Katz DP (1997). Applied nutrition in ICU patients: A consensus statement of the American College of Chest Physicians. Chest.

[24] Fraser IM (1986). Effects of refeeding on respiration and skeletal muscle function. Clin Chest Med.

[25] Reigner J, Mercier E, Le Gouge A, Boulain T, Desachy A, Bellec F, Clavel M, Frat JP, Plantefeve G, Quenot JP, Lascarrou JB, Clinical Research in Intensive Care and Sepsis Group (2013). Effect of not monitoring residual gastric volume on risk of ventilator-associated pneumonia in adults receiving mechanical ventilation and early enteral feeding: a randomized controlled trial. JAMA.

[26] McClave SA, Taylor BE, Martindale RG, Warren MM, Johnson DR, Braunschweig C, McCarthy MS, Davanos E, Rice TW, Cresci GA, Gervasio JM, Sacks GS, Roberts PR, Compher C, Society of Critical Care MedicineAmerican Society for Parenteral and Enteral Nutrition (2016). Guidelines for the Provision and Assessment of Nutrition Support Therapy in the Adult Critically Ill Patient: Society of Critical Care Medicine (SCCM) and American Society for Parenteral and Enteral Nutrition (A.S.P.E.N.). JPEN J Parenter Enteral Nutr.

[27] Singer P, Blaser AR, Berger MM, Alhazzani W, Calder PC, Casaer MP (2019). ESPEN guideline on clinical nutrition in the intensive care unit: European Society for Parenteral and Enteral Nutrition (ESPEN). Clin Nutr..

[28] Montejo JC, Miñambres E, Bordejé L, Mesejo A, Acosta J, Heras A (2010). Gastric residual volume during enteral nutrition in ICU patients: the REGANE study. Intensive Care Med.

[29] Linn DD, Beckett RD, Foellinger K (2015). Administration of enteral nutrition to adult patients in the prone position. Intensive Crit Care Nurs.

